# Intestinal Decontamination Therapy for Dyskinesia and Motor Fluctuations in Parkinson's Disease

**DOI:** 10.3389/fneur.2021.729961

**Published:** 2021-09-10

**Authors:** José Fidel Baizabal-Carvallo, Marlene Alonso-Juarez, Robert Fekete

**Affiliations:** ^1^Department of Sciences and Engineering, University of Guanajuato, León, Mexico; ^2^Department of Medicine, National Polytechnic Institute, México City, Mexico; ^3^Department of Neurology, New York Medical College, Valhalla, NY, United States

**Keywords:** parkinsonism, microbiota, Parkinson's disease, dysbiosis, treatment, dyskinesia, motor fluctuations

## Abstract

Parkinson's disease is neurodegenerative disorder with an initial robust response to levodopa. As the disease progresses, patients frequently develop dyskinesia and motor fluctuations, which are sometimes resistant to pharmacological therapy. In recent years, abnormalities in gut microbiota have been identified in these patients with a possible role in motor manifestations. Dysbiosis may reduce levodopa absorption leading to delayed “On” or “no-On” states. Among 84 consecutive patients with PD, we selected 14 with levodopa-induced dyskinesia and motor fluctuations with a Movement Disorders Society Unified Parkinson's Disease Rating Scale (MDS-UPDRS) part IV ≥ 8 points following a trial of pharmacological adjustment 2–3 months prior to study enrollment or adjustments in deep brain stimulation therapy. Patients received treatment with sodium phosphate enema followed by oral rifaximin and polyethylene glycol for 7 and 10 days, respectively. Evaluations between 14 to 21 days after starting treatment showed improvement in MDS-UPDRS-IV (*P* = 0.001), including duration (*P* = 0.001) and severity of dyskinesia (*P* = 0.003); duration of medication “Off”-state (*P* = 0.004); functional impact of motor fluctuations (*P* = 0.047) and complexity of motor fluctuations (*P* = 0.031); no statistical improvement was observed in “Off” dystonia (*P* = 0.109) and total motor scores (*P* = 0.430). Marked to moderate improvement in dyskinesia was observed in 57% of cases with blinded evaluation of videos. About 80% of patients perceived moderate to robust improvement at follow-up. A therapeutic strategy aimed at decontamination of intestines showed benefit in motor fluctuations and dyskinesia. Further studies should confirm and clarify the mechanism of improvement observed in these patients.

## Introduction

Parkinson's disease (PD) is a neurodegenerative disorder characterized by progressive loss of dopaminergic neurons in the *substantia nigra* (SN). As the disease progresses the basal ganglia become progressively depleted of dopamine making supplementary dopaminergic treatment necessary to overcome motor symptoms (1). However, a substantial proportion of patients eventually develop motor fluctuations and levodopa-induced dyskinesia (LID) that may ultimately show a poor response to adjustments of dopaminergic medications and to anti-dyskinetic drugs leading clinicians to use invasive therapies such as deep brain stimulation (DBS) (1).

In recent years, a growing number of studies indicate that patients with PD have abnormal populations of gut microbiota along with small intestinal bacterial overgrowth (SIBO), conditions known as dysbiosis (2–4). Experimental studies in mice and clinical observations in patients with PD indicate that dysbiosis may be implicated in increased motor activity and motor fluctuations (5–7). One study showed that the prevalence of unpredictable motor fluctuations, such as longer “Off-time,” “delayed-On” and “no-On” state, were significantly higher in patients with SIBO and Helicobacter pylori infections, compared to controls (3). Moreover, improvement in motor fluctuations was observed following eradication of SIBO, in that study, but with high relapse rate at 6 months (3). Although emerging evidence links dysbiosis with the pathogenesis and pathophysiology of PD; there is a dearth of studies showing improvement in motor symptoms following therapies intended to decrease or modify the gut microbiota. A previous study showed that bowel cleansing could decrease intestinal microbiota load by 31-fold (8). We hypothesized that an intestinal decontamination therapy would decrease motor fluctuations in PD. We conducted an open-label study, but included blinded video-evaluations to assess the effect of a treatment strategy intended to decrease the load of intestinal bacteria in patients with PD suffering moderate to severe LID and motor fluctuations with limited response to pharmacological management.

## Materials and Methods

We studied consecutive patients diagnosed with PD according to the Queen Square Brain Bank Criteria (9) in a tertiary care center for movement disorders, from June 2018 to March 2020. Inclusion criteria considered patients aged 18-years old or older with moderate to severe dyskinesia and/or motor fluctuations defined by a Movement Disorders Society Unified Parkinson's Disease Rating Scale (MDS-UPDRS) part IV score equal or higher than eight points following pharmacological and/or deep brain stimulation adjustments. Pharmacological adjustments in time and dose of levodopa and other anti-parkinsonian agents such as entacapone, rasagiline, dopamine receptor agonists and amantadine was carried out 2–3 months prior to enrollment. The MDS-UPDRS-IV evaluates the duration and severity of dyskinesia, motor fluctuations and “Off” dystonia. Motor assessments were performed with part III of MDS-UPDRS prior to the next dose of levodopa to avoid interference by severe peak-dose dyskinesia. The International Parkinson and Movement Disorders Society provided written permission for the use of the MDS-UPDRS for the purpose of this study. Exclusion criteria considered patients with chronic renal failure, decompensated heart failure, colonic abnormalities precluding the use of evacuating enema, known allergies to prescribed medications to reduce microbiota load and patients who declined to participate in the study.

Additional evaluations included: (1) an abdominal X-ray in order to assess the fecal load by means of the Leech score (10) and (2) The Clinical Global Impressions Scale (CGIS) was used to evaluate the severity of dyskinesia, motor fluctuations and “Off” dystonia at baseline and improvement after therapy (11). A movement disorders physician performed all clinical evaluations, between 24 and 48 h prior treatment. The study was approved by the local committee of research and ethics of Torre Santé and it was carried out according to the declaration of Helsinki. Patients or a close family member provided written informed consent to participate in the study.

### Procedure

Patients were hospitalized during 4–5 h to receive two saline colon enemas with sodium phosphate Fleet®, 2 to 3 h apart, appropriate hydration was provided during the procedure. If signs of fecal impaction were observed, the stools were removed manually prior to the first enema. Patients were discharged with oral treatment consistent with rifaximin 200 mg three times a day for 7 days and polyethylene glycol-3350, 17 gr per day in water for 10 days, but it was continued if necessary. Importantly, no modifications in time and dose of dopaminergic therapy or anti-dyskinetic medication (i.e., amantadine) were done at baseline or during the study. Patients were instructed not to change their diet during the observation time following the study therapy.

### Outcomes

Patients were evaluated 14 to 21 days after the hospitalization day. Improvement in motor function was assessed with MDS-UPDRS-III scores, whereas the effect of the therapy on dyskinesia and motor fluctuations was assessed with the MDS-UPDRS-IV. Patients and family members rated the amount of amelioration with the “global improvement” part of the CGIS. Moreover, we carried out blinded evaluations of video recordings (M.A-J.) in order to assess improvement or worsening of dyskinesia. Videos were presented in a random order (at baseline and at follow-up), and the evaluator had to decide whether the patient was pre- or post-therapy. We classified the follow-up state according to this scale: 3: markedly better, 2: moderately better, 1: mildly better, 0: no changes up-to−3 markedly worse.

Additionally, we contacted patients or family members at 3-months for follow-up and asked them to rate their level of perceived improvement by the CGIS “global improvement.” The scale ranges from 1: markedly better, 2: moderately better, 3: mildly better or 4: no changes up-to 7: much worse.

## Statistics

Data were summarized in percentages, means and standard deviations. The paired *t* test was used to compare mean values of MDS-UPDRS part III and IV scores at baseline and at follow-up, as these values showed appropriate symmetry in the distribution assessment. Whereas the non-parametric Wilcoxon signed rank test was used to compare individual MDS-UPDRS-IV sub-scores at baseline and at follow-up. All statistical evaluations were performed using SPSS version 22, *P*-values below 0.05 were considered significant.

## Results

**Figure 1:**
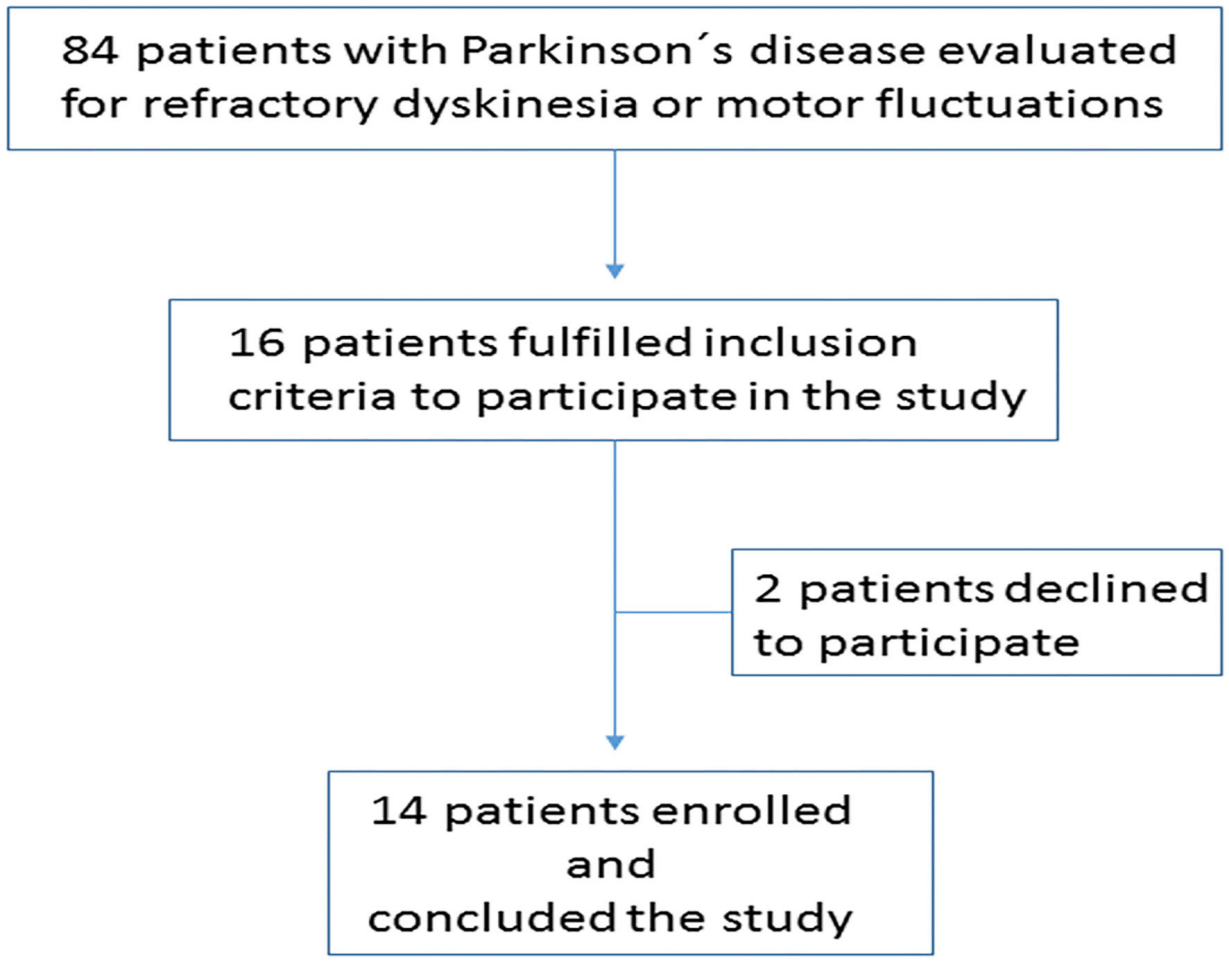
Cohort attrition.

**Figure 2:**
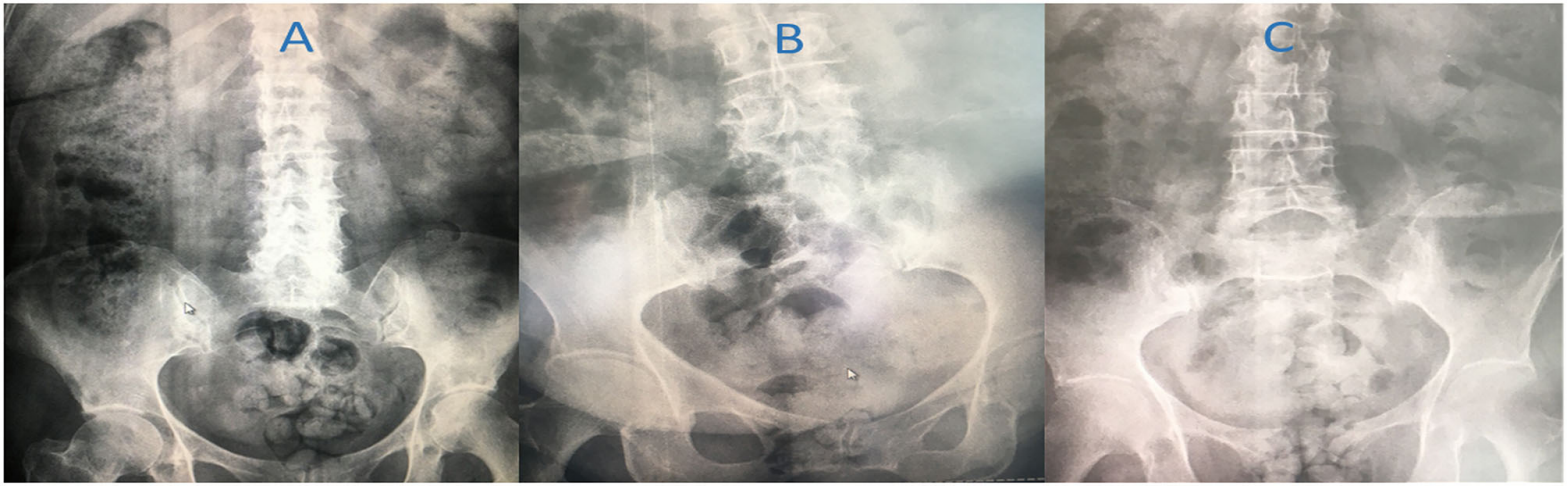
Examples of fecal retention in this cohort. **(A)** severe fecal retention with impaction in a 68-year-old woman with motor fluctuations reporting moderate improvement following therapy, **(B)** pan-colonic fecal retention in an 80 year-old woman with severe dyskinesia, improving following therapy (see Supplementary Video 2); **(C)** fecal retention with rectal distention in 69-year old woman with dyskinesia who had moderate improvement following therapy.

Among 84 consecutive patients with PD, 16 fulfilled the inclusion criteria, two patients declined to participate (Figure 1). A total of 14 patients (11 females), aged 69.43 ± 11.05 years were enrolled in the study, baseline features are presented in Table 1. All patients had moderate to severe fecal retention on abdominal x-rays with frequent fecal impaction (Figure 2). There were two patients treated with DBS, one with bilateral subthalmic nucleus and one with bilateral globus pallidus internus. Both patients underwent DBS therapy due to severe motor fluctuations 3 and 4 years before enrollment in this study. They had an estimated 70% improvement following DBS surgery. In both cases, patients developed severe dyskinesia or motor fluctuations, despite extensive revision of stimulation parameters and configurations. DBS-induced dyskinesia was ruled out after turning-off the devices for 1 h and not noticing changes in the clinical state.

**Table 1:** Summary of clinical features of included patients.

	**Mean ± SD**
Age at evaluation (years)	69.43 ± 11.05
Evolution time of PD (years)	12.7 ± 10.06
LEDD (mg/day)	1,199 ± 870
**X-ray abdomen**	
Leech score^a^	11.57 ± 5.0
**CGIS-improvement (following treatment)**	
At 14 days	1.92
At 3 months	2.35
	No. (%)
**Type of dyskinesia**	
Peak-dose	10 (71.4)
Diphasic	11 (78.6)
Both	7 (50)
**CGIS-severity (at baseline)**	
Moderate	4 (28.6)
Marked	10 (71.4)
**CGIS-improvement (following treatment)**	
Much better	5 (35.7)
Moderately better	6 (42.9)
Mildly better	2 (14.3)
No change	1 (7.1)
**Blinded evaluations of videos for dyskinesia**	
Markedly better	5 (35.7)
Moderately better	3 (21.4)
Mildly better	3 (21.4)
No change	2 (14.2)
**Patients reporting some improvement in:**	
Time on dyskinesia	14 (100)
Functional impact of dyskinesia	11 (78.6)
Time on “Off” state	10 (71.4)
Functional impact of fluctuations	6 (42.8)
Complexity of motor fluctuations	7 (50)
Painful dystonia in “Off” state	3 (21.4)

*CGIS, clinical global impressions scale; PD, Parkinson's disease; LEDD, levodopa equivalent daily dose. A Leech score ranges from 0 to 15, higher score means worse constipation*.

No improvement in the MDS-UPDRS part III (motor scores) was detected in any patient at follow-up; however, improvement in scores concerning duration and severity of dyskinesia, duration in the medication “Off” state and complexity of motor fluctuations was observed; whereas no statistically significant improvement was observed in the medication “Off” dystonia at follow-up (Table 2). In blinded evaluations of video-recording, five patients were classified as markedly better, three were moderately better, three mildly better and 2 had no change (Table 1). All patients were correctly classified at baseline or at follow-up. No patient was classified as worsening following therapy. One patient had his video missing for assessment.

**Table 2:** Comparison of scores related to the movement disorders society unified Parkinson's disease rating scale parts III and IV items, before and after treatment.

**Item**	**At Baseline**	**At follow-up**	**Percentage of improvement**	***P*-value**
Part III Total score	53.43 ± 23.8	52.86 ± 23.4	1.07	0.430
4.1. Time on dyskinesia	2.57 ± 1.4	0.86 ± 0.8	66.5	0.001
4.2. Functional impact of dyskinesia	2.64 ± 1.4	0.93 ± 0.9	64.7	0.003
4.3.Time in the “Off” state	2.50 ± 1.1	1.43 ± 0.6	42.8	0.004
4.4. Functional impact of motor fluctuations	3.00 ± 1.1	2.36 ± 1.0	21.3	0.047
4.5. Complexity of motor fluctuations	2.14 ± 1.2	1.43 ± 0.7	33.2	0.031
4.6 Painful dystonia in the “Off” state	1.21 ± 1.5	0.79 ± 1.1	34.7	0.109
Part IV Total score	14.07 ± 4.4	7.79 ± 3.4	44.6	0.001

Moderate to marked improvement in dyskinesia or motor fluctuations between 14 and 21 days after treatment were perceived by 11 (80%) patients according to the CGIS mean score: 1.92 (lower scores mean higher improvement). At 3-months, 9 (64%) patients still perceived moderate to marked improvement in dyskinesia or motor fluctuations, mean score: 2.35. No case of outburst dyskinesia or motor fluctuation was observed following therapy. Transitory diarrhea was the only reported side effect occurring in 5 (36%) of cases; it was treated adjusting the daily dose of polyethylene glycol-3350. Examples of clinical improvement are presented in Supplementary Videos 1–3.

## Discussion

In this study, we observed that patients with moderate to severe dyskinesia and motor fluctuations resistant to pharmacological adjustments, improved following a therapeutic strategy intended to intestinal decontamination. These findings suggest that fecal retention, which leads to bacterial overgrowth and contributes to dysbiosis, may have a potential role in the pathogenesis of LID and motor fluctuations. This is important, because severe constipation has shown a prevalence of 7% in patients with PD and of 20% in patients with a parkinsonian syndrome (12). In our experience, however, the frequency of bowel movement may be misleading for fecal load, as some patients with fecal impaction or coprostasis may have paradoxic diarrhea or overflow evacuations (13). In our study, we observed a high frequency of severe fecal retention in our patients by X-ray examination.

The composition of gut microbiota is usually affected immediately following bowel-cleansing preparation, with some studies showing lasting effect (14). However, the composition of gut microbiota tended to return to baseline levels after 14 days in other studies (8, 15). Colonic lavage with polyethylene glycol is associated with changes in luminal as well as mucosa-adherent bacteria with variable degrees of change (16). We used rifaximin a luminal, well-tolerated antibiotic with uncommon adverse effects and high effectivity to treat SIBO (17, 18). A meta-analysis concluded that rifaximin has shown to be safe and effective in patients with SIBO with eradication rates above 70% (19).

The mechanism of improvement in LID and motor fluctuations in our patients is unclear. If intestinal decontamination increases the absorption of levodopa, then peak-dose dyskinesia would be anticipated following the proposed therapy; however, such effect was not seen, instead a decrease in LID and motor fluctuations were observed. A study in 6-OHDA-lesioned hemiparkinsonian rats treated with pulsatile administration of levodopa showed appearance of dyskinesia when exposed to lipopolysaccharide (LPS), an endotoxin derived from gut microbiota (6). Increased release of TNF-α by lymphocytes stimulated by LPS may contribute to dyskinesia by promoting maladaptative synaptic plasticity in corticostriatal synapses (20). Moreover, blockage of the striatal IL-1β receptor and the anti-inflammatory corticosterone have shown a dose-dependent reduction in dyskinesia in experimental parkinsonian rats (21). LPS may stimulate the expression of nitric oxide (NO) synthase in the CNS, increasing the levels of NO, contributing to dyskinesia (20). It is unclear, however, whether such effects also occur in humans.

An association between SIBO and motor fluctuations may also exist, as shown in a Chinese study of 182 patients with PD (7). Improvement in motor fluctuation was also observed in our study. Emerging evidence has shown that intestinal bacteria *Enterococcus faecalis* has a conserved tyrosine decarboxylase able to metabolize levodopa, accelerating its gut metabolism (22). Whether the use of a luminal antibiotic may promote a more consistent intestinal absorption of levodopa, decreasing fluctuations is a plausible hypothesis that requires further testing.

Some studies have shown improvement in motor scores and function following diverse strategies for gut microbiota manipulation. A recent randomized trial reported that probiotic consumption 8 × 10^9^ vs. placebo, was related to a decrease in 4.8 points in the MDS-UPDRS score, along with a decrease in C-reactive protein, malondialdehyde and insulin levels; whereas insulin resistance improved and glutathione levels increased (23). Another studied based on dietary changes and bowel cleansing with daily fecal enemas for 8 days showed improvement in motor scores along with reduced requirements of dopaminergic medication in patients with PD (24). Microbiota diversity was associated with lower motor scores, although repetitive enemas showed reduction of Clostridiaceae bacteria (24). These findings add evidence that manipulation of gut microbiota in PD can hasten the motor and improve systemic inflammatory markers. Improvement can also occur in patients undergoing DBS therapy that developed severe dyskinesia and motor fluctuations as observed in two of our patients.

Our study has limitations, including the lack of randomization and relatively low number of cases studied (*n* = 14). It is unclear which of the used measure had the major impact in the clinical manifestations of our patients; however, patients reported major improvement within the first 48 to 72 h following colon enema, suggesting that decreasing the fecal load in the colon may provide the most prominent effect. Studies have shown that the metabolic activity in distal colon can match that of the human liver; making this part of the colon a major source of bacterial endotoxins and other metabolic byproducts that may gain access to systemic circulation due to increased permeability of the colonic barrier, a process known as “leaky-gut” syndrome (25). A lack of comparison arm is a major limitation of this study that should be addressed in a further trial. A randomized trial using dyskinesia improvement as an outcome of 65% would require 22 subjects, 11 per arm to demonstrate a statistically significance difference, considering a statistical power of 0.8 and alpha error of 0.05. Another limitation is that we did not test microbiota before and after the treatment. Previous studies have shown inconsistent results in bacteria modification after bowel preparation that may depend on the underlying microbiota in each individual in addition to the type and intensity of the bowel cleaning therapy (14, 15, 26). Plasma levels of levodopa before and after treatment would also provide insights into the effect of the proposed therapy in levodopa absorption and should be considered in further studies.

## Conclusions

Here, we present a simple strategy that seems to improve moderate to severe LID and motor fluctuations in patients suffering PD with unsatisfactory response to pharmacological adjustments. This therapy could be implemented in patients waiting for or with contraindication for more definitive therapies such as DBS; but can also provide benefit in patients already undergoing this therapy. Further studies should confirm these findings and define the mechanism of improvement for these patients.

## Data Availability Statement

The raw data supporting the conclusions of this article will be made available by the authors, under reasonable request.

## Ethics Statement

The studies involving human participants were reviewed and approved by Torre Medica Santé comité de investigación y ética. The patients/participants provided their written informed consent to participate in this study. Written informed consent was obtained from the individual(s) for the publication of any potentially identifiable images or data included in this article.

## Author Contributions

JB-C: conception, organization, execution the research project, and writing of the first draft and review and critique the manuscript. MA-J organization and execution the research project and writing of the first draft manuscript. RF: review and critique the manuscript. All authors contributed to the article and approved the submitted version.

## Conflict of Interest

The authors declare that the research was conducted in the absence of any commercial or financial relationships that could be construed as a potential conflict of interest.

## Publisher's Note

All claims expressed in this article are solely those of the authors and do not necessarily represent those of their affiliated organizations, or those of the publisher, the editors and the reviewers. Any product that may be evaluated in this article, or claim that may be made by its manufacturer, is not guaranteed or endorsed by the publisher.

## Supplementary Material

Supplementary Video 1 This a 74-year-old man with PD of long evolution. He developed severe peak of dose of dyskinesia (segment 1) following an episode of acute constipation requiring hospitalization. After colon enema and luminal antibiotic there is a remarkable improvement in dyskinesia (segment 2).

Supplementary Video 2 This is an 80-year-old woman with PD of 7 years of evolution. She has prominent dyskinesia and motor fluctuations of about 1 year of evolution. Segment 1 is at baseline, 3 h after the last dose of levodopa; it shows prominent dyskinesia. Segment 2 is 3 h after the last dose of dopaminergic therapy.

Supplementary Video 3 This is 65-year-old woman with PD, since about 16 years; she has prominent diphasic dyskinesia. Segment 1 is at baseline 4 h after the last dose of levodopa and amantadine. Segment 2 is 14 days after starting therapy; 4 h after the last dose of unchanged medications; there is marked improvement in diphasic dyskinesia.
